# Investigating neonatal health risk variables through cell-type specific methylome-wide association studies

**DOI:** 10.1186/s13148-024-01681-3

**Published:** 2024-05-22

**Authors:** Thomas L. Campbell, Lin Y. Xie, Ralen H. Johnson, Christina M. Hultman, Edwin J. C. G. van den Oord, Karolina A. Aberg

**Affiliations:** 1 Center for Biomarker Research and Precision Medicine, Virginia Commonwealth University, 1112 East Clay Street, P. O. Box 980533, Richmond, VA 23298-0581 USA; 2 Department of Medical Epidemiology and Biostatistics, Karolinska Institutet, Stockholm, Sweden

**Keywords:** Neonatal, Biomarker, Methylation, Gestational age, Apgar, Jaundice, Pre-term

## Abstract

Adverse neonatal outcomes are a prevailing risk factor for both short- and long-term mortality and morbidity in infants. Given the importance of these outcomes, refining their assessment is paramount for improving prevention and care. Here we aim to enhance the assessment of these often correlated and multifaceted neonatal outcomes. To achieve this, we employ factor analysis to identify common and unique effects and further confirm these effects using criterion-related validity testing. This validation leverages methylome-wide profiles from neonatal blood. Specifically, we investigate nine neonatal health risk variables, including gestational age, Apgar score, three indicators of body size, jaundice, birth diagnosis, maternal preeclampsia, and maternal age. The methylomic profiles used for this research capture data from nearly all 28 million methylation sites in human blood, derived from the blood spot collected from 333 neonates, within 72 h post-birth. Our factor analysis revealed two common factors, size factor, that captured the shared effects of weight, head size, height, and gestational age and disease factor capturing the orthogonal shared effects of gestational age, combined with jaundice and birth diagnosis. To minimize false positives in the validation studies, validation was limited to variables with significant cumulative association as estimated through an in-sample replication procedure. This screening resulted in that the two common factors and the unique effects for gestational age, jaundice and Apgar were further investigated with full-scale cell-type specific methylome-wide association analyses. Highly significant, cell-type specific, associations were detected for both common effect factors and for Apgar. Gene Ontology analyses revealed multiple significant biologically relevant terms for the five fully investigated neonatal health risk variables. Given the established links between adverse neonatal outcomes and both immediate and long-term health, the distinct factor effects (representing the common and unique effects of the risk variables) and their biological profiles confirmed in our work, suggest their potential role as clinical biomarkers for assessing health risks and enhancing personalized care.

**Supplementary Information:**

The online version contains supplementary material available at 10.1186/s13148-024-01681-3.

## Background

Adverse neonatal outcomes are a prevailing cause of infant mortality and morbidity worldwide. For example, preterm birth (≤ 36 weeks) is the number one cause of neonatal death with nearly 1 million deaths annually [1]. Likewise, low Apgar scores (< 7) double the risk of neonatal mortality, neonatal infections, asphyxia related complications, respiratory distress, and neonatal hypoglycemia compared to that of healthy score (> 7) [2]. These adverse outcomes not only effect short-term health and mortality but have also been associated with an increased risks of chronic health conditions later in life [3, 4].

Neonatal outcomes are often correlated suggesting that in addition to measuring unique aspects of neonatal health, they share common risk factors [5, 6]. Furthermore, they are often “multifaceted” where different aspects of the same neonatal outcome may reflect different biological mechanisms. Birth weight, for example, is determined by various components, including bone density, internal organ mass, muscle development, adipose tissue, and fluid levels, that each can be influenced by distinct regulatory mechanisms. For example, it has been suggested that fat-mass reflects the intra-uterine environment, whereas fat-free mass is more likely to be altered by genetic factors [7]. As the different aspects of the same measure may have different clinical relevance, it is critical to take the correlated and multifaceted nature of neonatal outcomes into account.

Studying epigenetic variation presents an avenue for better understanding how adverse neonatal outcomes contribute to health consequences later in life and presents a novel way to validate the biological relevance of the common and unique effects that make up these outcomes. DNA methylation (DNAm), one of the most commonly studied epigenetic mechanisms, entails the addition of methyl groups to DNA’s cytosine-phosphate-guanine (CpG) dinucleotides [8, 9]. In most tissues, including blood, DNA methylation occurs almost exclusively at the 28 million CpG sites in the human genome. Changes in DNAm may be the result of genetic, environmental, and developmental factors. DNAm plays a pivotal role in regulating gene activity [10] and, as the function of cells differs, DNAm patterns are often cell-type specific. However, when assessing DNAm in whole tissues, the measurements encompass contributions from all cell-types present [11]. Therefore, cell-type specific analyses are needed to identify effects from individual cell-types.

In this study, we aimed to improve traditional assessment of neonatal outcomes by performing factor analysis to identify both common and unique effects underlying common neonatal health risk variables. Furthermore, we apply criterion-related validity testing through methylome-wide association study (MWAS), by leveraging sequence-based methylation profiles from neonatal blood, encompassing nearly all 28 million sites in the human genome. MWASs are performed both on whole blood (i.e., bulk tissue) and, by using a statistical deconvolution approach, on each cell-type specifically. To further characterize the validation results, we performed comprehensive Gene Ontology (GO) analyses on the associated genes.

## Methods

### Neonatal study samples and methylomic data

All 333 participants were part of a larger Swedish cohort [12]. Participants were born in Sweden from 1975 to 1989 and were of Swedish descent (defined as having both parents born in Sweden). For the current study, we use neonatal blood spots along with neonatal health information reported in Swedish national registries. Blood spots were collected within 72 h of birth by the Swedish hospital system for routine neonatal newborn screening. Neonatal health risk variables included gestational age calculated from the mother’s first day of last period, Apgar score assessed one minute after birth and three measures indicating body size (birth weight, birth height, and head size). Furthermore, we obtained information of the presence of jaundice, maternal preeclampsia diagnosis, the maternal age at delivery, and whether any disease diagnosis for the child was made at birth. The study was approved by institutional review boards in Sweden and at Virginia Commonwealth University.

Methylome-wide profiles from each neonatal blood sample were recently generated using an optimized procedure for methyl-binding domain enrichment sequencing (MBD-seq) [13], that assesses nearly all CpGs in the human genome [14–16]**.** Following strict quality control on CpGs and reads, we retained 24,244,667 commonly methylated autosomal CpGs that were assayed by an average of 46.3 million (SD = 5.9 million) reads per sample.

### Main analysis in four steps

**Fig. 1:**
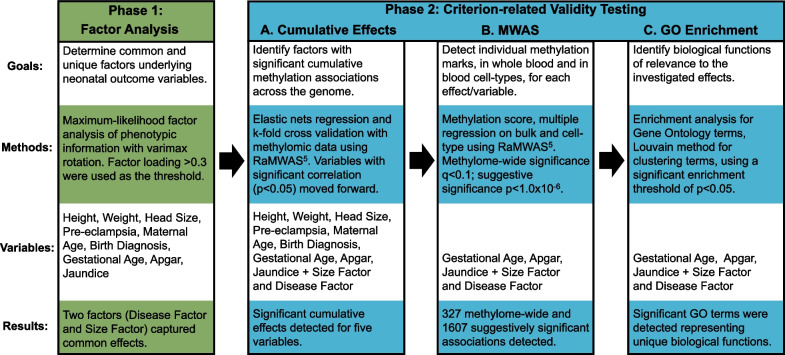
Overview of the Study Design. Each of the two phases, and steps, of the analysis are summarized with respect to goals, methods, variables, and results. Abbreviations: MWAS, Methylome-wide Association Study; GO, Gene Ontology

As depicted in Fig. 1, to detect common and unique factors across the neonatal health risk variables, we performed a first phase where we used exploratory factor analysis on the intercorrelations among the nine risk variables. Next, to study the criterion-related validity of the identified factors, we applied a second phase involving three steps. First, in step A, we screened for the presence of nominally significant (*P* < 0.05) cumulative effects of associated methylation sites in whole blood, to determine which factors and variables to include in the full-scale analysis, thereby minimizing false positive findings in the downstream analysis. These analyses were performed using the methylation-risk score (MRS) function in RaMWAS, a flexible analysis package specifically designed to handle large-scale methylation datasets [17]. In step B, for each neonatal risk variable, with nominal significance in the screening step, we performed a full-scale MWAS in whole blood and for each cell-type using RaMWAS [17].

Association tests are performed using linear regression while accounting for selected covariates including sex, lab-technical variables, and cell-type proportions. Additionally, for risk variables loading high (> 0.3) on the size and/or disease factors, the factors were also included as covariates, which allows for detection of unique effects by removing common effects. Furthermore, as previously described [13], the first two principal components from the methylation data were used as covariates to address unmeasured confounders. Cell-types investigated were B cells, monocytes, granulocytes, natural killer (NK) cells, cytotoxic T (cT) cells, and T helper (Th) cells. The cell-type specific MWASs were performed using a statistical deconvolution approach that has been carefully described and evaluated previously [18]. The method was introduced over 20 years ago, is widely used in gene expression studies [19–21] and has been applied to DNA methylation studies by us and others [22–25]. In short, the cell-type proportions in combination with the statistical deconvolution algorithm are applied to disentangle the association with the neonatal health risk variable, for each cell-type [18, 26]. The statistical model for the cell-type specific analyses is:

$$Y^{{{\text{bulk}}}} = { }\mathop \sum \limits_{c = 1}^{{n_{{\text{c}}} }} m_{{\text{c}}} P_{{\text{c}}} + { }\mathop \sum \limits_{c = 1}^{{n_{{\text{c}}} }} m_{c}^{{{\text{RV}}}} \left( {{\text{RV }} \times { }P_{{\text{c}}} } \right) + E$$

Thus, measurements from bulk tissue $Y^{{{\text{bulk}}}}$ are regressed on $c = 1$ to $n_{{\text{c}}}$, cell-type proportions $P_{{\text{c}}}$, and the product of the risk variable (RV) by cell-type proportions $\left( {{\text{RV}} \times P_{c} } \right)$. The model allows for covariates (not shown) and residual effects $E$. Coefficient $m_{{\text{c}}}$ is the effect of cell-type $c$. The parameter $m_{c}^{RV}$ is used to test the null hypothesis that there is no association for cell-type $c$ and the health risk variable.

To account for multiple testing and declare methylome-wide significance in the MWASs, we controlled the false discovery rate (FDR) at the 0.1 level [27]. Suggestively significant methylation associations were defined as *P* < 1.0 × 10^–6^. Finally, in step C, enrichment analysis for GO terms, using ConsensusPathDB-human release 35[28], were performed for each risk variable, with all genes linked to suggestively significant (*P* < 1.0 × 10^–6^) findings in the bulk and cell-type specific MWASs [28, 29]. Methodological details are presented in Additional file 1.

## Results

### Neonatal study samples

One participant was born prematurely (< 32 weeks) and was excluded from all further analyses. For three participants the birth records indicated gestational age exceeding 301 days (43 weeks), i.e., exceeding the length of a normal pregnancy [30]. These values were interpreted as likely clerical errors and were set to missing. In addition, missing information occurred for gestational age, birth weight, birth height, and head size for a total of 12 of the 2,988 total number of assessments (0.4% missing) across the different neonatal risk variables. To allow for inclusion of participants despite the missing values (no individual had more than two missing values), missing values were imputed with the MICE R-package, using available information from gestational age, birth weight, birth height, head size and sex [31]. Table 1 provides information about the study sample and the investigated neonatal health risk variables.

**Table 1:** Summary of Data for the 332 Participants Included in the Statistical Analyses

Variables	Post-imputation		Missing before imputation
	N	%	
Males	191	57.5	0
Jaundice	17	5.1	0
Birth Diagnosis	99	29.8	0
Preeclampsia	24	7.2	0

	Mean	SD	
Gestational age (days)	278	12.6	5
Apgar score	8.9	1.02	0
Birth weight (grams)	3,490	526	1
Birth height (cm)	50.1	2.43	3
Birth head size (cm)	34.5	1.68	3
Maternal age (years)	27.4	4.83	0
B cells	0.08	0.03	0
Granulocytes	0.39	0.08	0
Monocytes	0.18	0.03	0
Nature killer cells	0.05	0.02	0
Cytotoxic T cells	0.15	0.03	0
T helper cells	0.14	0.04	0

N, number of participants; SD, standard deviation

### Phase 1: factor analysis

The results of the factor analysis revealed two distinct factors **(**Table 2 and Additional file 1: Figure S1), size factor and disease factor, capturing the shared effect of the risk variables. The variables with high loading (> 0.3) on the size factor were weight, head size, height, and gestational age. Similarly, disease factor captured orthogonal shared effects with high loadings, including gestational age, jaundice, and birth diagnosis. Pair-wise correlations between each risk variables are shown in Additional file 1: Table S1.

**Table 2:** Factor loadings and cumulative effects of associated methylation sites

Neonatal health risk variables	Factor loadings		Cumulative Effects	
	size factor	Disease factor	correlation	*P*
Gestational age	**0.561**	**− 0.418**	0.132	**8.09 × 10**^**–3**^
Weight	**0.954**	0.075	− 0.050	8.18 × 10^–1^
Head size	**0.735**	− 0.102	− 0.024	6.68 × 10^–1^
Height	**0.818**	0.140	0.075	8.55 × 10^–2^
Maternal age	0.034	0.200	0.046	2.03 × 10^–1^
Apgar	0.160	− 0.140	0.126	**1.10 × 10**^**–2**^
Jaundice	− 0.197	**0.645**	0.202	**1.12 × 10**^**–4**^
Preeclampsia	0.031	0.103	0.039	2.41 × 10–^1^
Birth diagnosis	− 0.207	**0.460**	− 0.045	7.94 × 10^–1^
Size factor	NA	NA	0.148	**3.42 × 10**^**–3**^
Disease factor	NA	NA	0.244	**3.78 × 10**^**–6**^

Variables loading high (> 0.3) on the factors are shown in bold. Spearman correlation is reported between risk variables and the assessed cumulative effect. *P* is the corresponding *p*-value. *P* < 0.05 is indicated in bold

### Phase 2A: cumulative association signal

As shown in Table 2, when assessing each of the now eleven neonatal variables, we observed significant cumulative effects for five variables. Both common effect factors showed highly significant Spearman correlations with the bulk methylation data: size factor (*r* = 0.24, *P* = 3.78 × 10^–6^); disease factor (*r* = 0.15, *P* = 3.42 × 10^–3^). Furthermore, significant correlations were observed for unique effects (after regressing the common effects) for gestational age (*r* = 0.13 *P* = 8.09 × 10^–3^) and jaundice (r = 0.20 *P* = 1.12X10^−4^) as well as for Apgar score (*r* = 0.13 *P* = 1.10 × 10^–2^). No significant correlations were detected for the individual size variables (birth weight, birth height, and head size), preeclampsia, maternal age, or birth diagnosis.

### Phase 2B: methylome-wide association studies (MWASs)

We performed full-scale MWASs, including bulk and cell-type specific analyses, for size factor, disease factor, gestational age and Apgar, and full-scale robust MWAS for jaundice, i.e., the variables for which we observed significant cumulative effects. Quantile–quantile plots and corresponding lambdas for full-scale MWAS, are shown for each risk variable in Additional file 1: Figure S2. The shape of these plots and the observed lambdas (0.932–1.076), as well as the lambdas from the robust MWAS (0.957–1.082) showed no signs of test statistic inflation, confirming the accuracy of our P values. An overview of the number of methylome-wide significant and suggestive findings, for each risk variable, are shown in Table 3. Here we confine ourselves to top methylome-wide significant (*q* < 0.1) findings for each cell-type and loci where multiple CpGs are linked to genes. Full results are provided in Additional file 2: Tables S2–Additional file 6: Table S6.

**Table 3:** Number of Methylome-wide/suggestively significant MWAS Findings (*q* < 0.1/*P* < 1.0 × 10^–6^)

Neonatal health risk variables	Bulk	B cells	Granulocytes	Monocytes	NK cells	cT cells	Th cells
Size Factor	13/49	5/94	10/45	12/100	0/37	27/100	0/32
Disease Factor	2/109	0/50	0/24	2/29	0/40	28/104	216/234
Gestational Age	0/37	0/59	0/32	0/18	0/17	0/8	0/15
Apgar	10/97	0/53	1/89	0/33	1/24	0/52	0/44
Jaundice	0/6	0/0	0/0	0/0	0/5	0/0	0/0

NK, natural killer; cT, cytotoxic T; Th, T helper

In regards to the common factors, for the size factor, we identified a total of 67 methylome-wide significant findings in bulk (*N* = 13), B cells (*N* = 5), granulocytes (*N* = 10), monocytes (*N* = 12) and cT cells (*N* = 27). The most significant findings for both bulk (*P* = 1.61 × 10^–10^, *q* = 0.003) and B cells (*P* = 3.23 × 10^–9^, *q* = 0.078) were intergenic, without any linked genes. In contrast, the most significant finding for granulocytes was linked to the BLNK (*P* = 3.35 × 10^–9^, *q* = 0.069), a gene involved in kinase signaling and previously associated with Alzheimer disease [32]. Of note, the same CpG, in the *BLNK* gene, that was detected in granulocytes was also the most significant finding in monocytes (*P* = 1.12 × 10^–11^, *q* = 0.0003), with the opposite direction of effect. This reinforces the importance of cell-type specific analysis to detect associations otherwise undetectable in bulk tissue. The most significant finding for cT cells was linked to the *ZNF131* gene (2 CpGs, *P* = 1.66 × 10^–9^–8.05 × 10^–9^, *q* = 0.022–0.027), found to be critical for T cell growth and development [33]. For disease factor, we identified a total of 248 methylome-wide significant findings, including bulk (*N* = 2), monocytes (*N* = 2), cT cells (*N* = 28), and Th cells (*N* = 216). The most significant findings for bulk (*P* = 5.51 × 10^–9^, *q* = 0.070), monocytes (*P* = 6.46 × 10^–10^, *q* = 0.016) and cT cells (*P* = 6.35 × 10^–10^, *q* = 0.015) were intergenic. The most significant finding in Th cells was linked to *UBR3* (P = 3.47 × 10^–11^, *q* = 0.0008), which has been suggested as a candidate gene for developmental delay in children [34].

For Apgar, we identified a total of 12 methylome-wide significant findings, including bulk (*N* = 10), granulocytes (*N* = 1), and NK cells (*N* = 1). The most significant finding for bulk was linked to the gene *MAN1A2* (*P* = 1.96 × 10^–9^, *q* = 0.015), a susceptibility gene for biliary atresia in neonates[35]. The significant CpG for granulocytes (*P* = 3.10 × 10^–9^, *q* = 0.075) was intergenic but the significant CpG in NK cells overlapped with *ZNF502* (*P* = 3.88 × 10^–9^, *q* = 0.094)*,* a possible biomarker for depression [36]. No methylome-wide significant findings were detected for the remaining unique effects of gestational age and jaundice, when the common effects were accounted for.

### Phase 2C: gene ontology enrichment analysis

The full results for the GO enrichment analysis and clustering, using suggestively significant MWAS findings, are presented in Additional file 7: Table S7. Here we confine ourselves to the most significant GO terms and the largest resulting clusters. The findings for size factor were enriched for 43 GO terms clustering into 7 groups (Additional file 1: Figure S3A). The most significant term was cytoskeleton (*P* = 4.00 × 10^–4^), which segregated to a cluster (green) involving cytoskeleton mitotic division. The largest cluster (blue) was centered on cell signaling and comprised 9 terms, the most significant being negative regulation of secretion by cell (*P* = 1.4 × 10^–2^). The results for disease factor showed enrichment of 30 terms that segregated into 8 cluster (Additional file 1: Figure S3B). Proteoglycan metabolic process was the most significant term (*P* = 2.5 × 10^–3^) and fell within a cluster (light green) relating to glycoprotein processes. The largest cluster (orange) involved 6 terms focused on nervous system development, the most significant being neuron projection development (*P* = 3.22 × 10^–2^).

In regard to unique effects, results for gestational age included 43 terms, grouped into 5 clusters (Additional file 1: Figure S3C). The most significant term was cell projection morphogenesis (*P* = 7.0 × 10^–4^), which was part of the largest cluster (green) containing 12 terms, centered on nervous system development. Findings for Apgar led to the enrichment of 22 terms segregated into 6 clusters (Additional file 1: Figure S3D). The most significant term was homophilic cell adhesion via plasma membrane adhesion molecules (*P* = 5.0 × 10^–4^), which lies with a cluster (red) centered on the components of the plasma membrane. The largest cluster (green) was involved in cell signaling and contained 6 terms, the most significant being intrinsic apoptotic signaling pathway (*P* = 1.8 × 10^–2^). Lastly, our findings for jaundice’s showed enrichment of 4 terms that clustered into 3 groups (Additional file 1: Figure S3E). The most significant term being negative regulation of biosynthetic process (*P* = 3.5 × 10^–3^), which fell within the only cluster (green) that contained more than 1 term.

## Discussion

Neonatal outcomes are often correlated and “multifaceted” constructs, suggesting they both, shared risk factors and different aspects of the same outcome may reflect different biological mechanisms. In this study, we performed factor analyses on a broad set of neonatal health risk variables to improve the assessment of neonatal outcomes by specifically distinguishing between the underlying common effects shared between multiple risk variables and unique effects specific for individual risk variables. We then investigated the criterion-related validity of these derived factors via methylation profiles from neonatal blood. Our analyses revealed two common and three unique effect factors with significant cumulative association signals. For these factors we observed multiple methylome-wide significant findings for specific genes of potential relevance and suggestive findings. This supports the biological validity of the derived factor model. Our results may prove of critical importance for future investigations and applications as different facets of the same neonatal risk factor may have different clinical correlates.

For example, we found highly significantly associated methylation differences when examining the common effects of gestational age via size factor and disease factor. This suggested biological effects of gestational age on DNA methylation are mediated through two common components, potentially involved in distinct functional mechanisms, represented by size factor and disease factor. To reinforce this validation the GO enrichment analysis unveils the involvement of nervous system development and cell signaling for both factors. However, a more intricate narrative emerges upon more detailed analysis of the results (i.e., GO terms and genes associated with each risk variable), while the broader biological processes seem similar, the finer branches within these processes diverge significantly across the risk variables. This highlights the importance of considering the correlated and multifaceted nature of neonatal outcomes.

In contrast to the findings related to gestational age, Apgar score did not load on either of the common factors and is solely composed of unique effects. This suggests, Apgar score should continue to be considered a distinct measure for assessing the immediate and long-term, health risks for neonates. In validation of this observation, we detected several highly significantly associated methylation sites when studying the Apgar score alone. The detection of these methylation marks further suggests the potential expanded valuable of this already important variable in a clinical setting.

The study results offer valuable insights into neonatal health risk variables. However, it is important to acknowledge potential limitations when interpreting the results. The current study involves samples from a relatively homogenous population, comprising individuals of Swedish descent whose biological and phenotypical information was collected via a standardized hospital system. While this study sample offers great homogeneity and consistency in data collection, it potentially limits the generalizability of the findings toward more diverse populations. In addition, the relatively limited sample sizes in this study, in particular for the analysis involving jaundice, which only include a small number of participants with this condition, leads to limited statistical power, which may result in that some methylation differences go undetected. However, we note that despite low statistical power, significant findings are detected.

Furthermore, our primary analyses, focusing on methylation marks associated with neonatal health risk variables in neonatal blood, are performed without consideration of any disorders the participants may develop later in life. However, it should be noted that the investigated participants were originally enrolled to study schizophrenia, a psychiatric disorder that typically manifests in late adolescents or early adulthood. Thus, the participants included 198 individuals that later in life developed schizophrenia and 135 individuals who, well into adulthood, had not been diagnosed with any psychiatric disorder. Because of this distribution of schizophrenia cases and controls it is feasible to further evaluate the potential influence of schizophrenia on the investigated risk variables and factor scores. Therefore, we performed confirmatory factor analyses. Results showed that the measurement structure is not significantly different (P = 0.06) suggesting the biological processes underlying the risk variables are similar across the two groups (Additional file 1).

Moreover, the current study sample involves limited information about the presence of prenatal exposures, for example, maternal smoking and prenatal infections, which could potentially cause confounding effects with the risk variables. Finally, we note the absence of nucleated red blood cells (nRBC) in our cell-type specific reference panel, a recognized limitation given nRBCs often are present the first few days after birth and, therefore, may contribute to the methylation profiles generated from whole blood [28, 29]. Despite these limitations, our results encourage future studies to further validate the derived factors. Future investigations of larger, and more diverse, cohorts will offer the potential to uncover further nuances of these findings.

## Conclusion

Our novel use of factor analysis showed that clinical neonatal outcomes are not unidimensional variables but rather multidimensional, effected by many underlying components. We successfully performed criterion-related validation analysis on neonatal health risk variables, using DNAm on a cell-type specific level, to show the derived factors have meaning and unique biological correlates. Our results may prove of critical importance for future investigations and clinical applications as the different risk factors may have different clinical correlates.

### Supplementary Information

Additional file1 (PDF 723 kb)

Additional file2 (XLSX 28 kb)

Additional file3 (XLSX 43 kb)

Additional file4 (XLSX 168 kb)

Additional file5 (XLSX 25 kb)

Additional file6 (XLSX 38 kb)

Additional file7 (XLSX 14 kb)

## Data Availability

The dataset analyzed during the current study is not publicly available due European Union Privacy Laws. RaMWAS is freely available from Bioconductor (https://bioconductor.org/packages/release/bioc/html/ramwas.html). The RaMWAS script used to perform cell-type specific association studies is available from GitHub: https://github.com/ejvandenoord/celltype_MWAS. In addition, R code to estimate the cell-type proportions by an empirical Bayes approach is also provided on GitHub: https://github.com/ejvandenoord/Empirical-Bayes-estimation-of-cell-type-proportions.

## Abbreviations

DNAm: DNA methylation
CpG: Cytosine-phosphate-guanine
MWAS: Methylome-wide association study
GO: Gene ontology
MBD-seq: Methyl-binding domain enrichment sequencing
MRS: Methylation risk score
NK: Natural killer cell
cT: Cytotoxic T cell
Th: T Helper cell
FDR: False discovery rate
