# Supplementary material for: Investigating neonatal health risk variables through cell-type specific methylome-wide association studies
Source: Clin Epigenetics. 2024 May 22;16:69. doi: 10.1186/s13148-024-01681-3 (PMC11112760; doi:10.1186/s13148-024-01681-3)
Supplement: Supplementary file 1 — Additional file1 (PDF 723 kb) [file 13148_2024_1681_MOESM1_ESM.pdf]

Supplementary material to:

Investigating Neonatal Health Risk Variables through Cell-type Specific Methylome-wide Association Studies

TABLE OF CONTENT

PHASE 1: FACTOR ANALYSIS 3

PHASE 2A: CUMULATIVE ASSOCIATION SIGNAL 3

PHASE 2B: BULK AND CELL-TYPE SPECIFIC METHYLOME-WIDE ASSOCIATION STUDIES (MWASS) 4

PHASE 2C: GENE ONTOLOGY ENRICHMENT ANALYSIS 5

RESULTS 6

EXPLORATORY FACTOR ANALYSIS 6

CONFIRMATORY FACTOR ANALYSIS 6

FIGURES 7

FIGURE S1. SCREE PLOT FOR EXPLORATORY FACTOR ANALYSIS 7

FIGURE S2. QQ PLOTS FOR METHYLOME-WIDE ASSOCIATIONS STUDIES 8

TABLES 17

TABLE S1. PAIR-WISE SPEARMAN CORRELATION BETWEEN THE NEONATAL HEALTH RISK VARIABLES 17

TABLE S2. SIZE FACTOR BULK AND CELL-TYPE MWAS RESULTS 18

TABLE S3. DISEASE FACTOR BULK AND CELL-TYPE MWAS RESULTS 18

TABLE S4. GESTATIONAL AGE BULK AND CELL-TYPE MWAS RESULTS 18

TABLE S5. APGAR SCORE BULK AND CELL-TYPE MWAS RESULTS 18

TABLE S6. JAUNDICE BULK AND CELL-TYPE MWAS RESULTS 18

TABLE S7. GO ENRICHMENT AND CLUSTERING RESULTS 18

REFERENCES 19

Methods

Phase 1: Factor Analysis

To detect the common effect factors in the neonatal health risk variables, we performed a maximum likely-hood exploratory factor analysis on the intercorrelations among the nine risk variables. A varimax rotation was performed to improve the interpretation of the factor solution. Factor scores were estimated by Bartletts method(1).

Phase 2A: Cumulative Association Signal

Before performing full-scale MWAS for whole blood and for specific cell types for the eleven risk variables (nine unique variables and two common effect factors), we screened for the presence of nominally significant cumulative effects of associated methylation sites in the bulk data, to minimize false positive findings. These analyses were performed using the methylation-risk score (MRS) function in RaMWAS, a flexible analysis package specifically designed to handle large-scale methylation datasets(2). To assess the cumulative effect, RaMWAS uses elastic nets regression and k-fold cross validation (k = 10) to obtain an unbiased estimate of the cumulative effect. The cumulative effect was measured by Spearman's correlation between the risk variable outcome predicted from the methylation data and the risk variable observed outcome. To avoid the exhaustive analysis of approximately 24 million CpGs, most of which are unlikely to be associated with the risk variable and may introduce irrelevant noise to the model, we opted to include only the top m sites from the MWAS in the MRSs, where m was 100, 250, 500, 1000, 2500, 5000, or 10000. This process was repeated for each of the k folds in a cross-validation approach. Importantly, the selection of the top m CpG sites and the estimation of their weights were conducted independently of the participants in the test set, ensuring an unbiased estimation of the cumulative association signal. For each value of m, we computed the correlation between MRS derived from the methylation data, and the risk

variable. The final model was selected based on the value of m where the correlation reached a stable plateau, as the rationale dictates that adding more markers should theoretically maintain or improve the power, though it may decline when an excessive number of irrelevant sites are included. For each neonatal risk variable with nominal significance ($P < 0.05$) we performed a full scale MWAS in whole blood and for each cell-type.

To control for confounding factors in these analyses we used sex, lab-technical variables, and cell-type proportions as covariates in the MWASs. In addition, for risk variables loading on any, or both, of either size factor and/or disease factor, the factors were also included as covariates. Including the factors as covariates allows for examination of the unique remaining effects, as the common effects contained in the factors are removed. As described previously(3), we also included the first two principal components from the methylation data as covariates in our analyses to account for unmeasured confounders in the methylation data.

Phase 2B: Bulk and Cell-type Specific Methylome-wide Association Studies (MWASs)

We performed MWAS of bulk using RaMWAS(2) and the covariates described in Phase 2A. Cell-type proportions were estimated directly from the methylation data from each sample, using an empirical Bayes estimator that substantially outperformed previous approaches(4). This approach uses recently created MBD-seq based neonatal reference panel that included profiles from the most common neonatal blood cell-types in neonates: B cells, granulocytes, monocytes, natural killer (NK) cells, cytotoxic T (cT) cells, and T-helper (Th) cells(3).

The cell-type specific MWASs were performed using a deconvolution approach where the cell-type proportions in combination with the statistical deconvolution algorithm are applied to disentangle the association signal for each cell-type(5, 6). QQ plots and lambda, calculated as the median of the observed results divided by the expected median of the null distribution. were evaluated for signs of test statistic inflation (**Figure S2).**

For dichotomized variables with few cases (<10%), the regular MWAS approaches described above may be unstable due to outliers. Therefore, a “robust MWAS approach”(7) was performed, which uses k-fold cross-validation (k=2), that was repeated 10 times with different seeds and then reports the average results.

Phase 2C: Gene Ontology Enrichment Analysis

Enrichment analysis for Gene Ontology (GO) terms using ConsensusPathDB-human release 35(8), were performed for each risk variable, with all genes linked to suggestively significant ($P<1.0x10^{-6}$) findings in the bulk and cell-type specific MWASs(8, 9). A gene was considered linked if the CpG was located within the body of the gene or within 10,000 bp upstream of the transcription start site. The analyses were performed with the R-package shiftR using 10,000 circular permutations(10), which effectively control the Type I errors even when highly correlated sites are present(11, 12), as is often the case with methylation data. Furthermore, because the permutations are performed on a CpG level, they take gene size (i.e., the number of CpGs per gene) into account, which further prevents bias. For each risk variable, nominally significant GO terms (P< 0.05), with at least five overlapping genes, were clustered based on overlapping gene content with the Louvain method for community detection for pathway clustering(13). Clustering plots are shown in **Figure S3.**

Results

Exploratory Factor Analysis

We employed scree plot analysis to determine the number of factors to include in the downstream analyses by visually inspecting the scree plot (**Figure S1**) and examining the eigenvalues associated with each factor(14). However, interpretations were inconclusive. Instead, we carefully examined the variables loading on each factor and found that only Apgar loaded on the third factor of the three-factor solution, which would be equivalent to analyzing the original variable. Based on these results a two-factor solution was chosen.

Confirmatory Factor Analysis

The lavaan R-package was utilized to test for measurement invariance across schizophrenia cases and controls, using the confirmatory factor analysis function to create the models(15). We then compared the constrained (i.e., "configural invariance") model, where the factor loadings and intercepts were constrained to be equal across groups, to the unconstrained model, where these parameters were allowed to vary freely.

The results showed the constrained model did not fit the data significantly poorer as compared to the unconstrained model (p=0.06), meaning the measurement structure is considered equivalent across groups, indicating that observed differences between the groups are not due to differences in measurement.

Figures

Figure S1. Scree Plot for Exploratory Factor Analysis

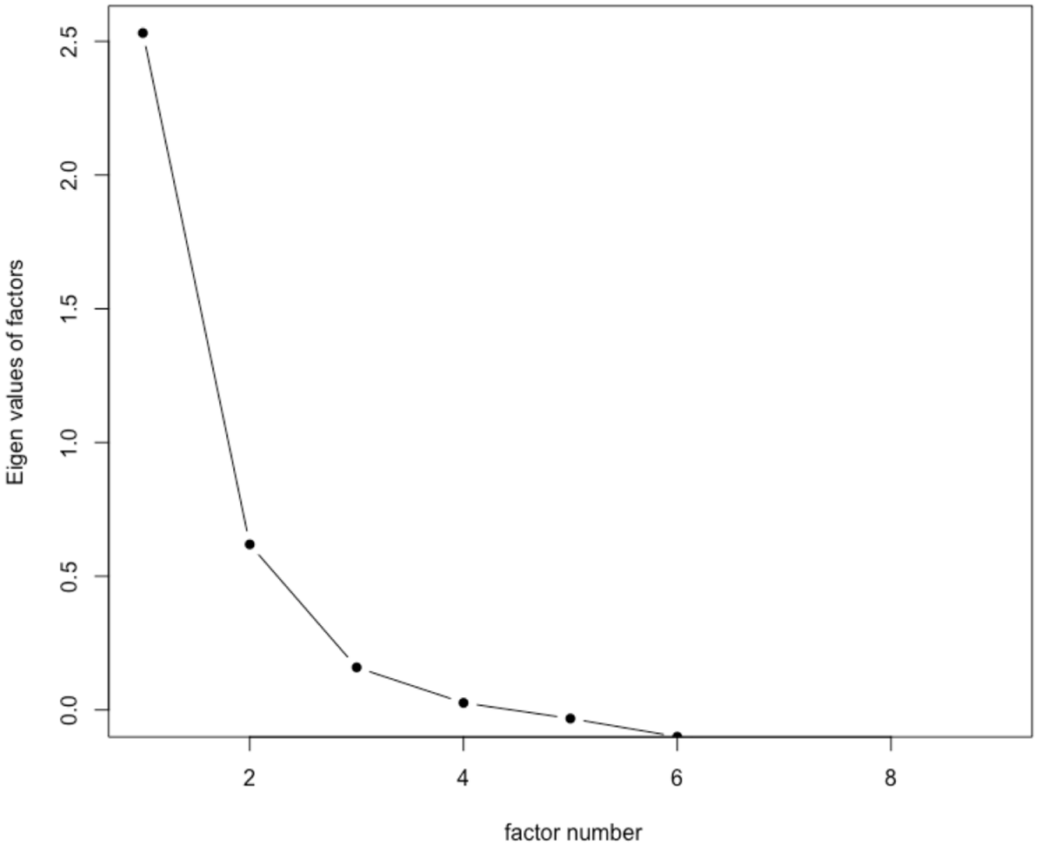

Scree plot of maximum-likelihood factor analysis for all nine neonatal risk variables (gestational age, Apgar score, birth weight, birth height, birth head size, jaundice, preeclampsia, maternal age, and birth diagnosis). Factor loadings are presented in the main text **Table 2**.

Figure S2. QQ Plots for Methylome-wide Associations Studies

A. Size Factor

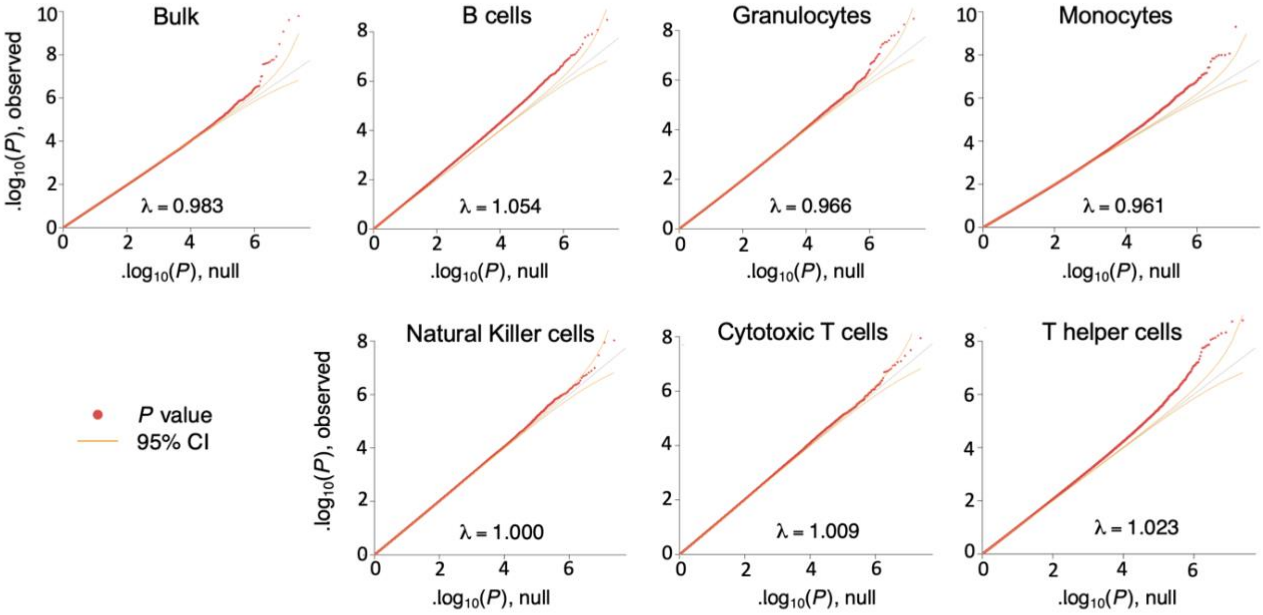

B. Disease Factor

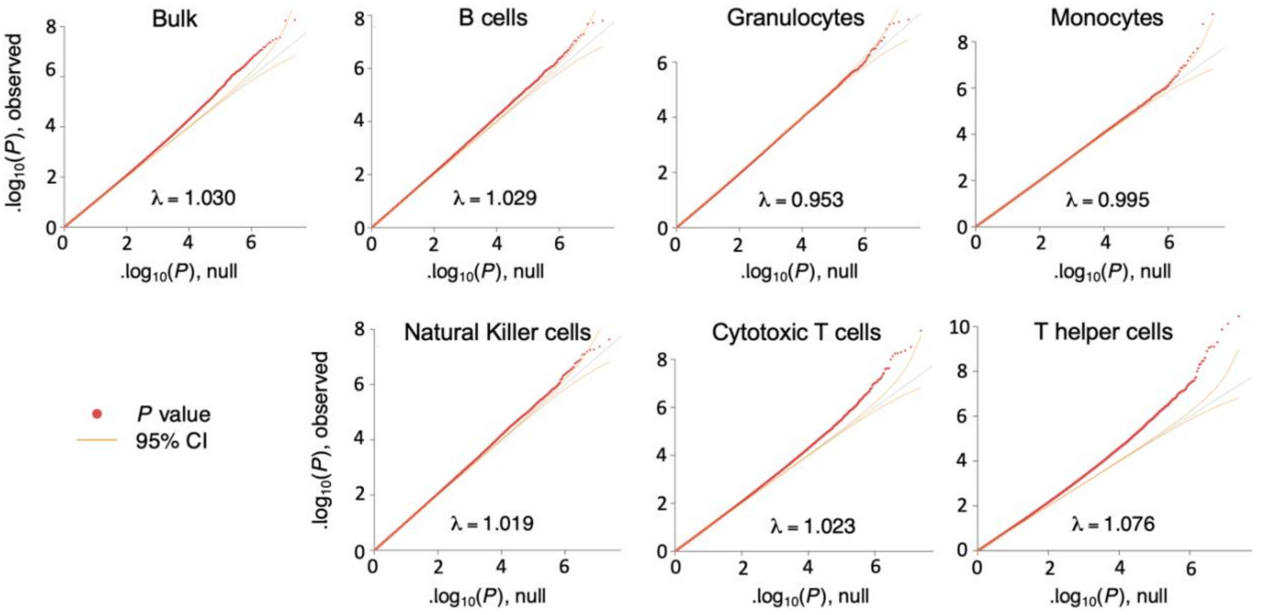

C. Gestational Age

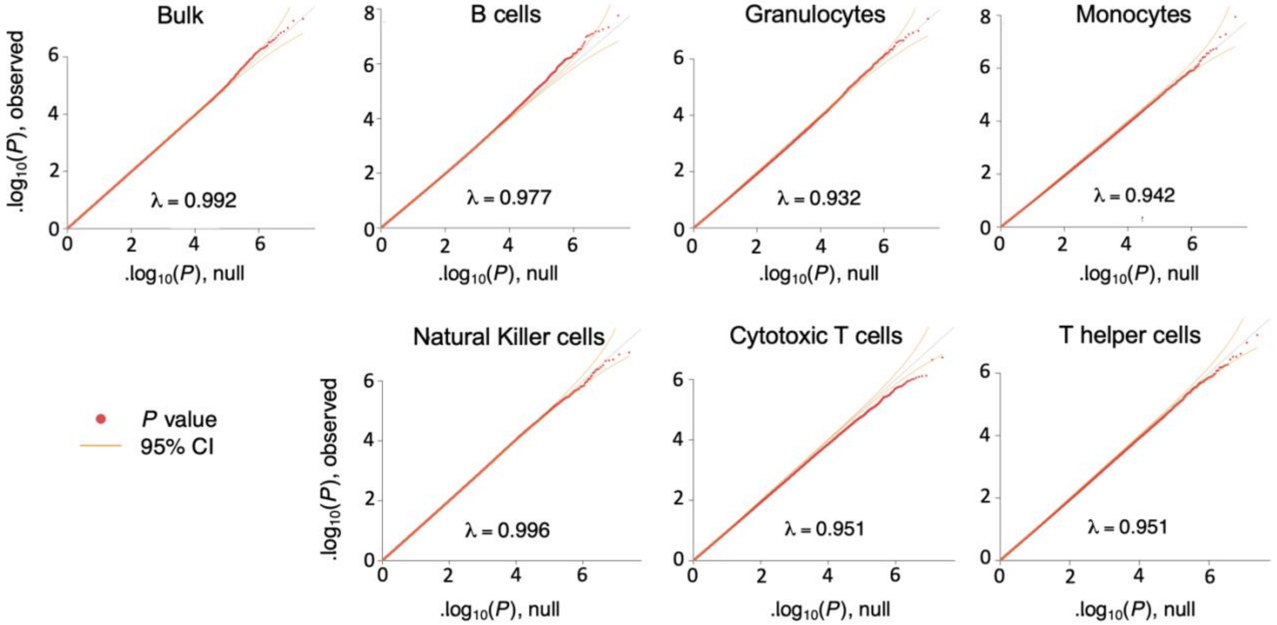

D. Apgar Score

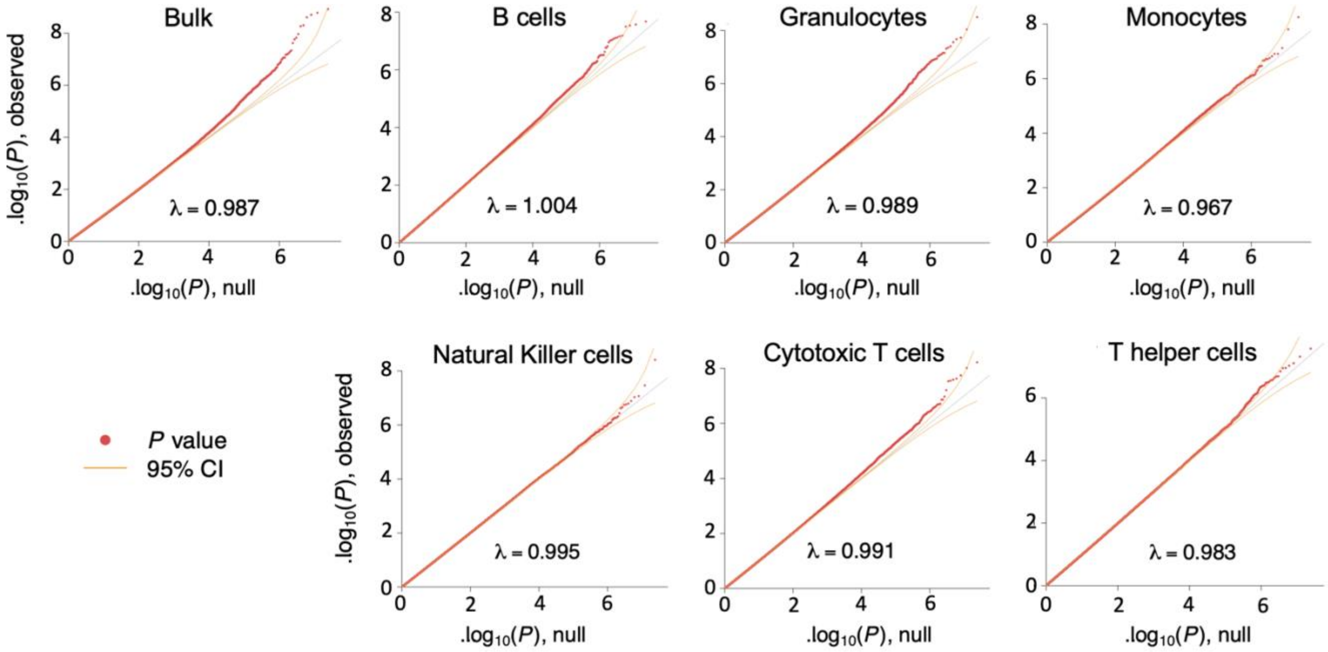

QQ plots and lambda for Methylome-wide Associations Studies for bulk and six cell-types. Y-axis shows the $\log_{10}(P)$ of the observed data and the X-axis displays the $\log_{10}(P)$ of the null. No QQ plots were generated for jaundice as the result from the robust MWAS represents the mean P value across 10 MWASs, which does not follow a uniform distribution so that under the null hypothesis markers are no longer expected to fall on the diagonal line in the QQ plot.

Figure S3. Significantly Enriched Gene Ontology Terms and Their Clustering

A. Size Factor

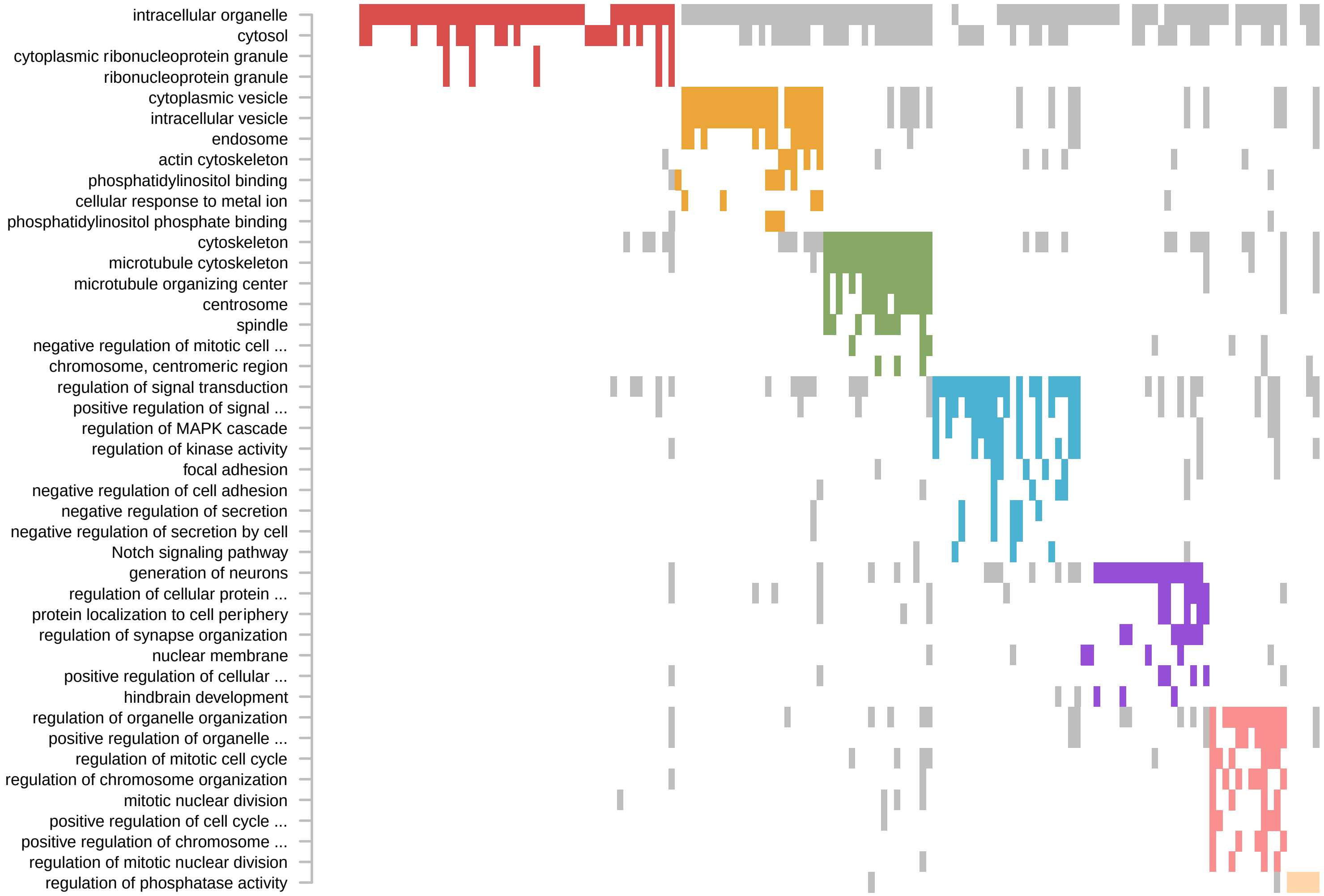

B. Disease Factor

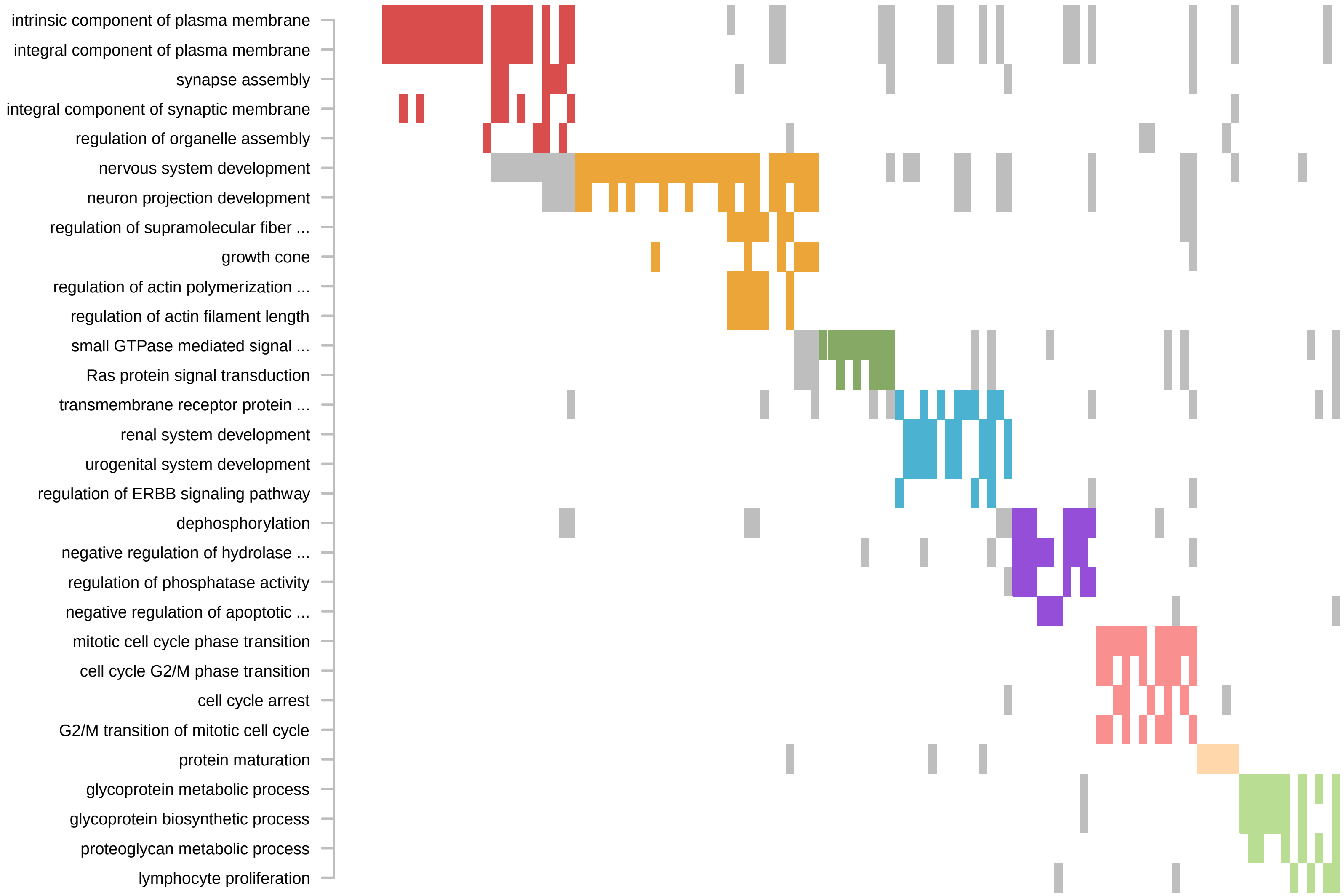

C. Gestational Age

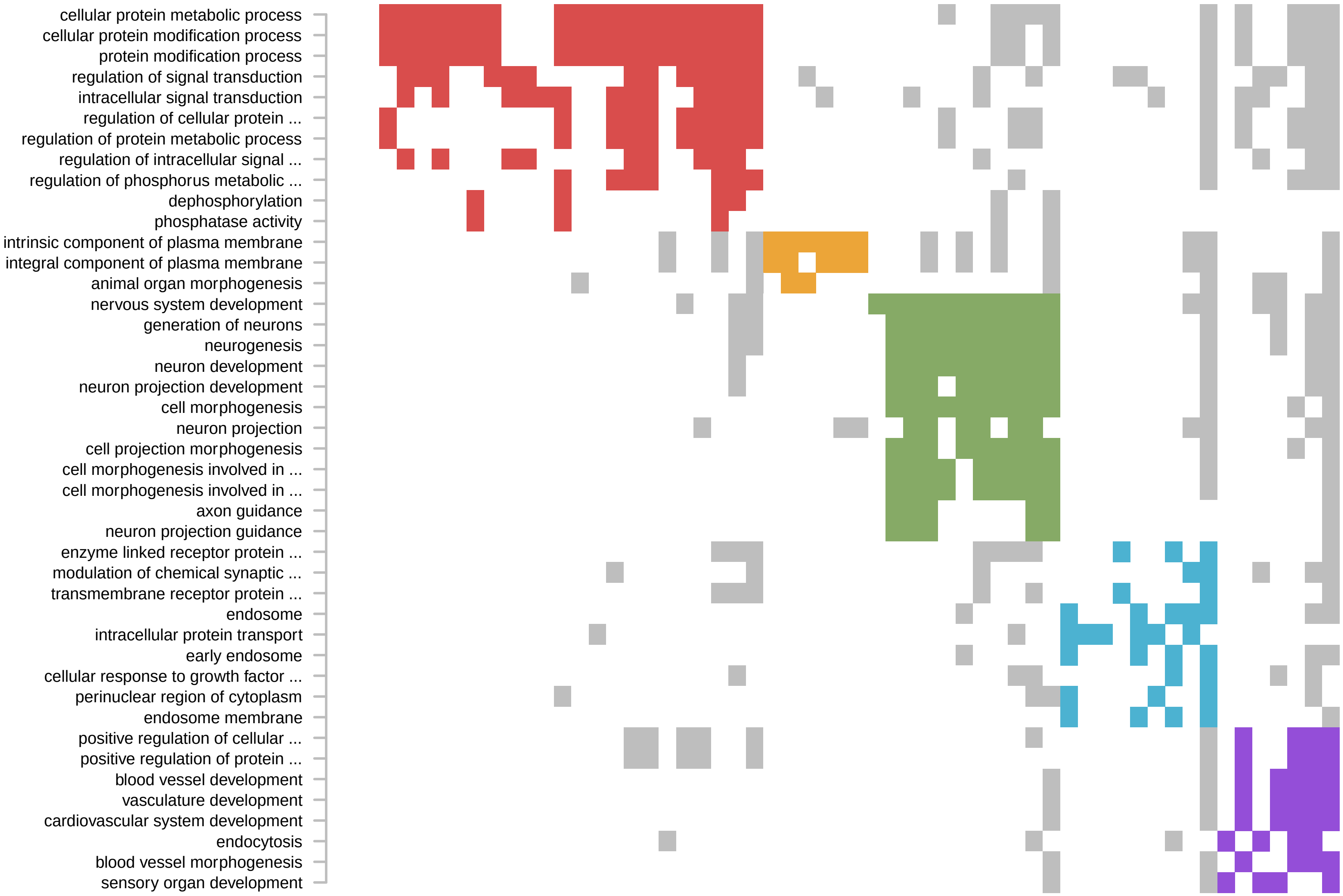

D. Apgar Score

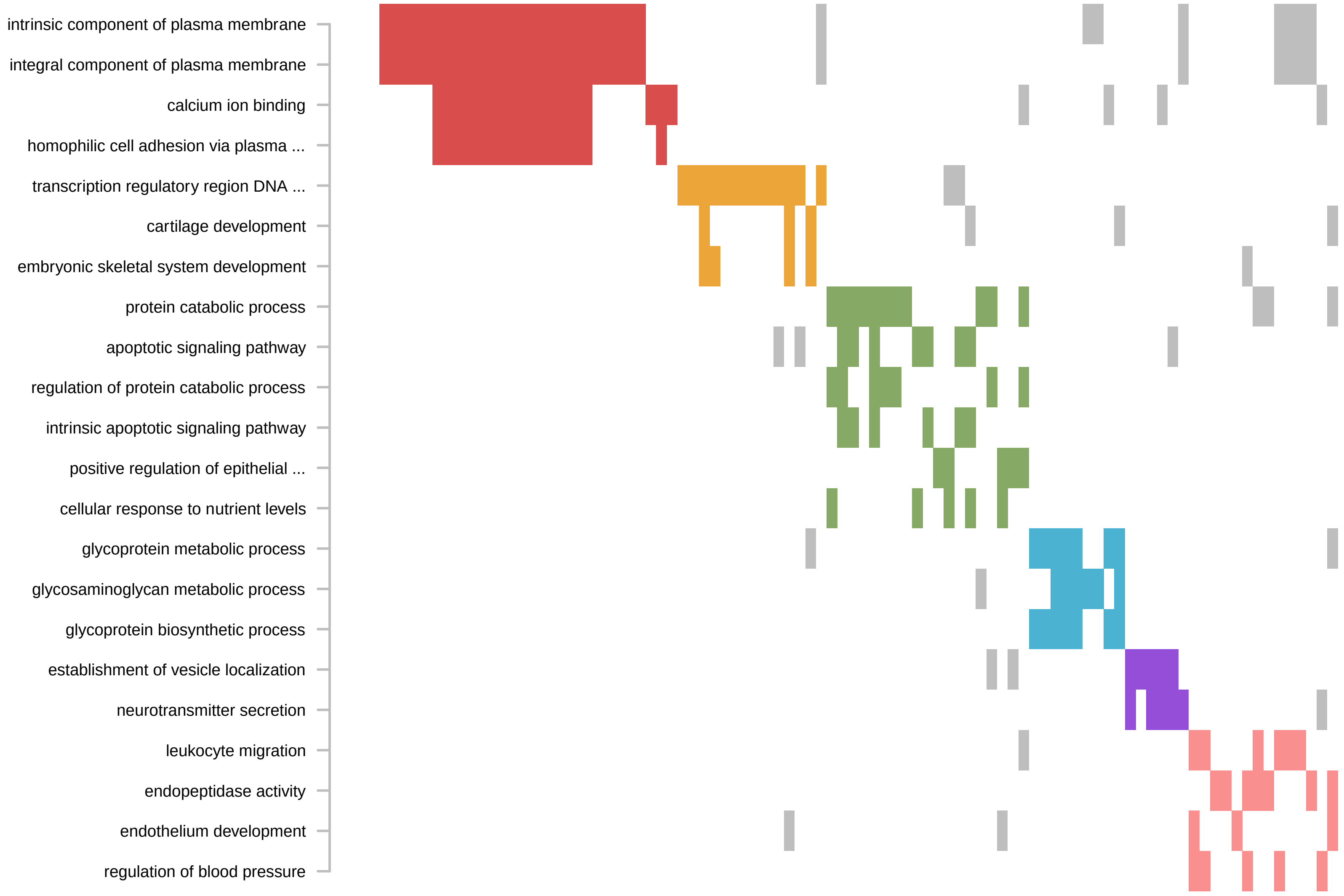

E. Jaundice

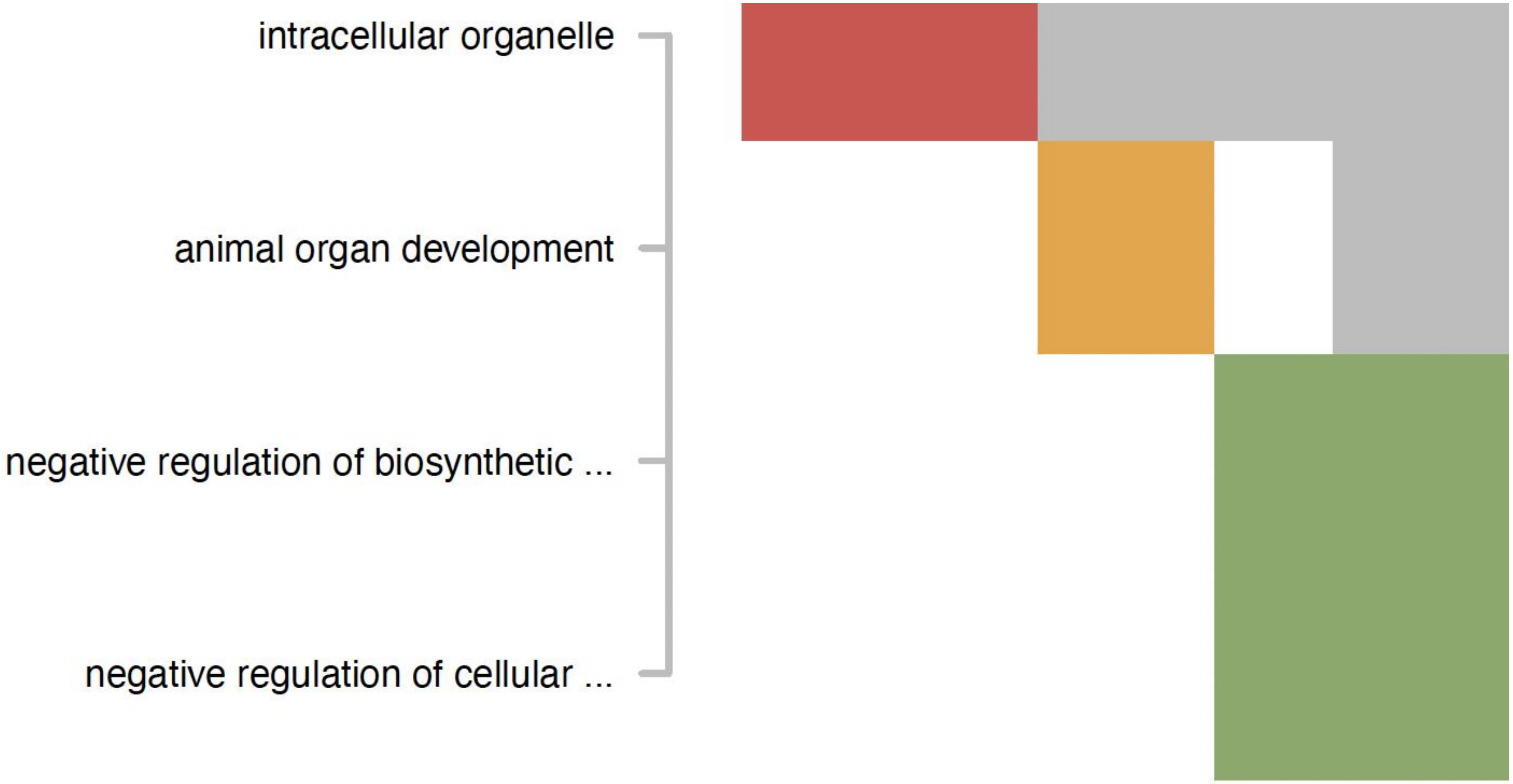
intracellular organelle
animal organ development
negative regulation of biosynthetic ...
negative regulation of cellular ...

Significantly enriched GO terms, for bulk and cell-type specific findings combined, for each risk variable. As GO terms often share genes, the raster plot visualizes the clustering of terms (y-axis) determined on the basis of their overlapping genes (x-axis). The solid rectangles indicate genes that both were among the suggestively significant ($P<1.0x10^{-6}$) methylome-wide association study results and were members of an enriched term. Only terms containing a minimum of five overlapping genes and those passing nominal significance ($P <0.05$) were retained. Full results are presented in **Table S7**.

Tables

Table S1. Pair-wise Spearman Correlation between the Neonatal Health Risk Variables

Risk Variables	Gestational Age	Weight	Head Size	Height	Mother's Age	Apgar Score	Jaundice	Preeclampsia	Birth Diagnosis
Gestational Age									
Weight	0.502								
Head Size	0.459	0.695							
Height	0.407	0.792	0.577						
Maternal Age	-0.085	0.045	-0.015	0.08					
Apgar Score	0.177	0.137	0.18	0.088	0.045				
Jaundice	-0.382	-0.143	-0.205	-0.065	0.119	-0.022			
Maternal Preeclampsia	-0.063	0.028	0.07	0.05	0.061	0.01	0.093		
Birth Diagnosis	-0.286	-0.159	-0.19	-0.127	0.053	-0.234	0.356	-0.055	

Table S2. Size Factor Bulk and Cell-type MWAS Results

See separate excel file: Campbell_Neonatal_Table_S2.xlsx

Table S3. Disease Factor Bulk and Cell-type MWAS Results

See separate excel file: Campbell_Neonatal_Table_S3.xlsx

Table S4. Gestational Age Bulk and Cell-type MWAS Results

See separate excel file: Campbell_Neonatal_Table_S4.xlsx

Table S5. Apgar Score Bulk and Cell-type MWAS Results

See separate excel file: Campbell_Neonatal_Table_S5.xlsx

Table S6. Jaundice Bulk and Cell-type MWAS Results

See separate excel file: Campbell_Neonatal_Table_S6.xlsx

Table S7. GO Enrichment and Clustering Results

See separate excel file: Campbell_Neonatal_Table_S7.xlsx

References

1. Bartlett MS. The statistical conception of mental factors. British journal of Psychology. 1937;28(1):97.

2. Shabalin AA, Hattab MW, Clark SL, Chan RF, Kumar G, Aberg KA, et al. RaMWAS: fast methylome-wide association study pipeline for enrichment platforms. Bioinformatics. 2018;34(13):2283-5.

3. van den Oord EJCG, Xie LY, Zhao M, Campbell TL, Turecki G, Kähler AK, et al. Genes implicated by a methylome-wide schizophrenia study in neonatal blood show differential expression in adult brain samples. Molecular Psychiatry. 2023.

4. van den Oord E, Aberg KA. Fine-grained cell-type specific association studies with human bulk brain data using a large single-nucleus RNA sequencing based reference panel. Sci Rep. 2023;13(1):13004.

5. Wang X, Park J, Susztak K, Zhang NR, Li M. Bulk tissue cell type deconvolution with multi-subject single-cell expression reference. Nat Commun. 2019;10(1):380.

6. Venet D, Pecasse F, Maenhaut C, Bersini H. Separation of samples into their constituents using gene expression data. Bioinformatics. 2001;17 Suppl 1:S279-87.

7. Oord EJvd, Guintivano JD, Aberg KA. A method to improve the reproducibility of findings from epigenome- and transcriptome-wide association studies. bioRxiv. 2023:2023.03.29.534761.

8. Gene Ontology C. The Gene Ontology resource: enriching a GOld mine. Nucleic Acids Res. 2021;49(D1):D325-D34.

9. Ashburner M, Ball CA, Blake JA, Botstein D, Butler H, Cherry JM, et al. Gene ontology: tool for the unification of biology. The Gene Ontology Consortium. Nat Genet. 2000;25(1):25-9.

10. Cabrera CP, Navarro P, Huffman JE, Wright AF, Hayward C, Campbell H, et al. Uncovering networks from genome-wide association studies via circular genomic permutation. G3 (Bethesda). 2012;2(9):1067-75.

11. Han LKM, Aghajani M, Clark SL, Chan RF, Hattab MW, Shabalin AA, et al. Epigenetic Aging in Major Depressive Disorder. Am J Psychiatry. 2018;175(8):774-82.

12. Aberg KA, Dean B, Shabalin AA, Chan RF, Han LKM, Zhao M, et al. Methylome-wide association findings for major depressive disorder overlap in blood and brain and replicate in independent brain samples. Mol Psychiatry. 2020;25(6):1344-54.

13. Blondel VD, Guillaume J-L, Lambiotte R, Lefebvre E. Fast unfolding of communities in large networks. Journal of Statistical Mechanics: Theory and Experiment. 2008;2008(10):P10008.

14. Cattell RB. The Scree Test For The Number Of Factors. Multivariate Behavioral Research. 1966;1(2):245-76.

15. Rosseel Y. lavaan: An R Package for Structural Equation Modeling. Journal of Statistical Software. 2012;48(2):1 - 36.
